# MEK and MCL-1 sequential inhibition synergize to enhance rhabdomyosarcoma treatment

**DOI:** 10.1038/s41420-022-00959-w

**Published:** 2022-04-07

**Authors:** Clara Alcon, Fernando Martín, Estela Prada, Jaume Mora, Aroa Soriano, Gabriela Guillén, Soledad Gallego, Josep Roma, Josep Samitier, Alberto Villanueva, Joan Montero

**Affiliations:** 1 Institute for Bioengineering of Catalonia (IBEC), Barcelona Institute of Science and Technology (BIST), 08028 Barcelona, Spain; 2 Networking Biomedical Research Center in Bioengineering, Biomaterials and Nanomedicine (CIBER-BBN), 28029 Madrid, Spain; 3 Developmental Tumor Biology Laboratory, Institut de Recerca Sant Joan de Déu, 08950 Esplugues de Llobregat, Spain; 4 Pediatric Cancer Center Barcelona (PCCB), Hospital Sant Joan de Déu Barcelona, 08950 Esplugues de Llobregat, Spain; 5 Group of Translational Research in Child and Adolescent Cancer, Vall d’Hebron Research Institute (VHIR), Universitat Autònoma de Barcelona (UAB), 08035 Barcelona, Spain; 6 Department of Surgery, Universitat Autònoma de Barcelona (UAB), 08193 Barcelona, Spain; 7 Department of Electronics and Biomedical Engineering, University of Barcelona (UB), 08028 Barcelona, Spain; 8 Program against Cancer Therapeutic Resistance (ProCURE), IDIBELL, Catalan Institute of Oncology, 08907 l’Hospitalet del Llobregat, Barcelona, Spain; 9 Xenopat S.L., Business Bioincubator, Bellvitge Health Science Campus, 08907 l’Hospitalet de Llobregat, Barcelona, Spain

**Keywords:** Targeted therapies, Paediatric cancer

## Abstract

Targeted agents have emerged as promising molecules for cancer treatment, but most of them fail to achieve complete tumor regression or attain durable remissions due to tumor adaptations. We used dynamic BH3 profiling to identify targeted agents effectiveness and anti-apoptotic adaptations upon targeted treatment in rhabdomyosarcoma. We focused on studying the use of BH3 mimetics to specifically inhibit pro-survival BCL-2 family proteins, overwhelm resistance to therapy and prevent relapse. We observed that the MEK1/2 inhibitor trametinib rapidly depleted the pro-apoptotic protein NOXA, thus increasing MCL-1 availability. Indeed, we found that the MCL-1 inhibitor S63845 synergistically enhanced trametinib cytotoxicity in rhabdomyosarcoma cells in vitro and in vivo. In conclusion, our findings indicate that the combination of a BH3 mimetic targeting MCL-1 with trametinib improves efficiency on rhabdomyosarcoma by blocking tumor adaptation to treatment.

## Introduction

Rhabdomyosarcoma (RMS) constitutes the most frequent form of soft tissue sarcoma during childhood, accounting for 5–8% of malignant tumors in children and adolescents [1–5]. RMS is classified based on histological characteristics into four different subtypes: embryonal (ERMS), alveolar (ARMS), spindle cell/sclerosing, and pleomorphic [5]. The two major subtypes are ARMS accounting for 20% and ERMS accounting for 60% of cases in children [1, 6]. ARMS commonly occurs in the extremities and has a high propensity for metastasis, while ERMS is more likely to present as localized disease in genitourinary or head/neck regions and have a better prognosis [6]. All risk-groups of RMS are treated with a multi-modal approach that includes chemotherapy, radiation, and surgery [7]. However, the cure rates for high-risk metastatic patients have not achieved significant progress in years [7]. RMS treatment continues to be based on combinations of conventional cytotoxic agents developed in the late 1960s [8] which are accompanied by therapy-related toxicities and a decrease in patients’ quality of life [9]. Targeted therapies have gained interest in the past years as an approximation to increase survival and decrease secondary effects in RMS patients [2, 8, 10, 11] mainly due to a better understanding of genetic and molecular alterations in patients’ tumors [1, 8, 10, 12]. Nevertheless, despite that targeted therapies have revolutionized treatment for some adult cancers [13] and pediatric hematological malignancies [14, 15], less significant progress has been achieved in pediatric solid tumors. Regarding RMS, preclinical studies have reported increased cytotoxicity when combining different targeted therapies with conventional chemotherapeutics [12], since single targeted agents alone would not be sufficient to reach clinical efficacy due to acquired resistances to those treatments [12].

Evasion of apoptosis represents a common feature of cancer persister cells that become resistant to treatments, and can be partially mediated by an increased expression of anti-apoptotic proteins [16, 17]. Apoptosis is a type of programmed cell death controlled by the BCL-2 family of proteins [18]. Members of this family are classified based on their structure, BCL-2 homology domains, and function [18, 19]. Alterations in different BCL-2 family members have been reported in RMS patients [2], including the overexpression of the anti-apoptotic proteins BCL-2 and MCL-1 [20, 21]. This could be of special interest to develop new therapeutic strategies targeting these anti-apoptotic proteins to treat high-risk or relapsed RMS patients [17, 22]. BH3 mimetics, small molecule inhibitors that mimic the action of sensitizer BH3-only proteins and selectively inhibit anti-apoptotic BCL-2 family members, are currently being exploited to overcome apoptosis resistance [19]. The functional assay dynamic BH3 profiling (DBP) can determine in less than 24 h how effective a treatment will be to engage apoptosis and it also allows us to identify cancer cells’ selective dependence on anti-apoptotic proteins to guide BH3 mimetics’ use and overcome therapy-induced resistance [19, 23–27, 28]. Specifically in RMS, using this approach we recently described novel rational combinations of chemotherapeutic agents with BH3 mimetics, that were effective both in vitro and in vivo [28]. Different molecular pathways implicated in the disease were identified as regulators of anti-apoptotic proteins’ transcription [2, 29]. However, therapeutic combinations of targeted agents with BH3 mimetics for RMS are understudied. We hypothesized that we could use DBP to evaluate the efficacy of targeted agents to determine treatment-associated anti-apoptotic adaptations and how to overcome them with BH3 mimetics.

## Results

### Identification of new potential targeted agents to treat RMS using DBP

**Fig. 1:**
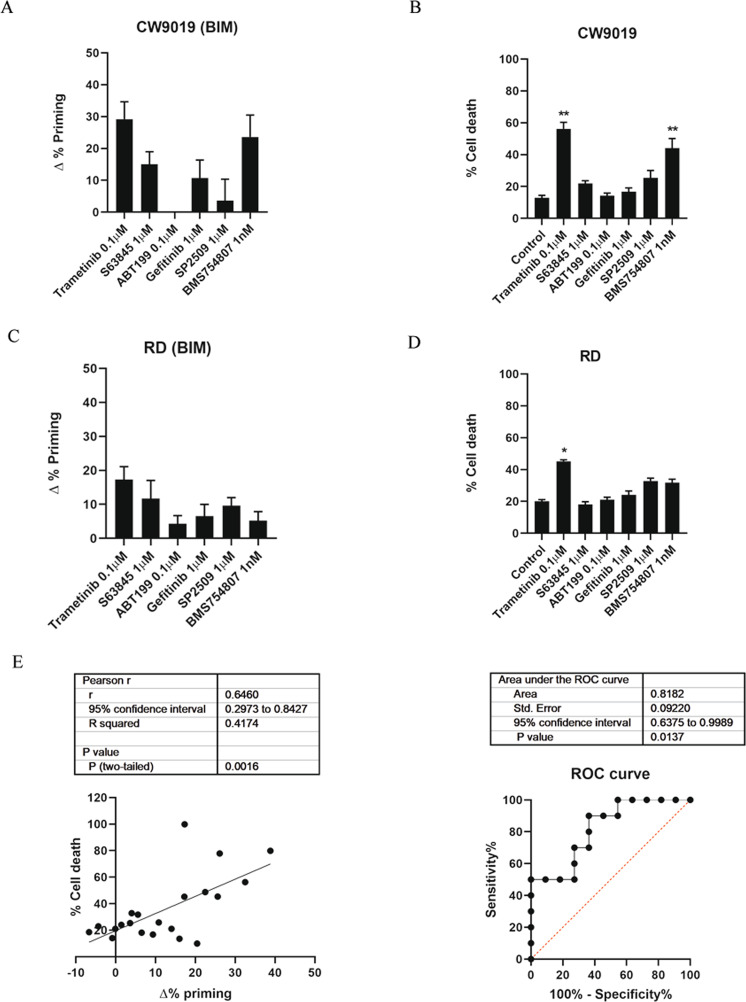
Dynamic BH3 profiling predicts sensitivity to targeted agents in CW9019 and RD cell lines. **A** Results from the DBP assay after 16 h incubation with treatments in CW9019 cells. Results expressed as ∆% priming represents the increase in priming compared to control cells. **B** Cell death results from Annexin V and propidium iodide staining and FACS analysis after 96 h incubation with the same targeted agents in CW9019 cells. **C** Results from the DBP assay after 16 h incubation with treatments in RD cells. Results expressed as ∆% priming represents the increase in priming compared to control cells. **D** Cell death results from Annexin V and propidium iodide staining and FACS analysis after 96 h incubation with the same targeted agents in RD cells. **E** Left plot showing the correlation between ∆% priming at 16 h and % cell death at 96 h. Receiver Operating Characteristic curve analysis showed at right. Values indicate mean values ± SEM from at least three independent experiments. ** *p* < 0.01 and * *p* < 0.05. All experiments were performed at least three times.

The discovery of oncogenes and tumor suppressors shed light on our understanding around molecular mechanisms leading to cancer [30, 31], aiming to improve treatment efficacy and reduce secondary effects in the clinic [32]. We used DBP to test some candidate targeted agents, that were chosen based on signaling pathways known to be altered in RMS [33], together with the BH3 mimetics S63845 (MCL-1 inhibitor) [34] and ABT-199 (BCL-2 inhibitor) [15] as increased BCL-2 and MCL-1 expression was previously reported in RMS patients [20, 21]. We observed an increase in ∆% priming upon 16 h of treatment with the MEK1/2 inhibitor trametinib [35] and the IGF-1R inhibitor BMS-754807 [36] in CW9019 cells (Fig. 1A). Moreover, trametinib also caused an increase in priming in RD cells (Fig. 1C). In contrast, RH4 cells showed a minor increase in ∆% priming after the treatment with the BH3 mimetics S63845 and ABT-199 and the EGFR inhibitor gefitinib [37]; and a higher response to the histone demethylase LSD1 inhibitor SP2509 [38] and to BMS-754807 treatments (Supplementary Fig. 1A). To determine the cytotoxic effectiveness of these targeted agents, we validated DBP predictions in vitro with Annexin V/propidium iodide (PI) cell death analyses by flow cytometry at 96 h. As shown, the most effective treatments identified by DBP in CW9019 cells after 16 h (trametinib and BMS-754807) correlated with the highest cytotoxicity (Fig. 1B). Furthermore, we could observe that the top treatments identified by DBP in RD and RH4 also caused the highest % of cell death assessed at 96 h (Fig. 1D and Supplementary Fig. 1B). When we statistically compared Δ% priming and % cell death in RMS cell lines, we observed a significant correlation between both measurements (Fig. 1E, left panel). It is important to highlight that neither S63845 nor ABT-199 induced significant cytotoxic effects as single agents in RMS cells (Fig. 1) which could be due to a cross-compensating effect of anti-apoptotic BCL-2 family proteins [39]. To determine DBP’s predictive capacity for targeted agents’ efficacy in RMS, we performed a Receiver Operating Characteristic (ROC) curve analysis [40]. Our results indicated an AUC of 0.81 (Fig. 1E, right panel), thus DBP was a good binary predictor for the subset of RMS cell lines and agents tested. The inhibition of MEK1/2 was previously reported as a potential molecular target for RMS [2, 10] and trametinib has been pointed as a potential therapeutic candidate in preclinical studies [12]. Moreover, the levels of IGF-1R have been associated with inferior survival rates of RMS patients [41] and the inhibition of the histone demethylase LSD1 [42] has been described as a potential molecular target for new therapies to treat RMS [12, 43, 44], matching our initial findings (Fig. 1).

### Combination of S63845 with MEK1/2 inhibitors to overcome therapy-induced resistance in RMS

Using DBP with specific BH3 peptides that mimic sensitizer BCL-2 family proteins we can identify which anti-apoptotic protein cancer cells employ to survive a specific treatment [19]. For instance, the contribution of BCL-2/BCL-xL could be measured using the BAD BH3 peptide, BCL-xL dependence with the HRK BH3 peptide and finally, MCL-1 dependence with the MS1 BH3 peptide [19, 45–48]. We can then determine how to overcome these adaptations using specific BH3 mimetics such as S63845 (MCL-1 inhibitor) [34], ABT-199 (BCL-2 inhibitor) [15] or A-1331852 (A-133) (BCL-xL inhibitor) [49].

**Fig. 2:**
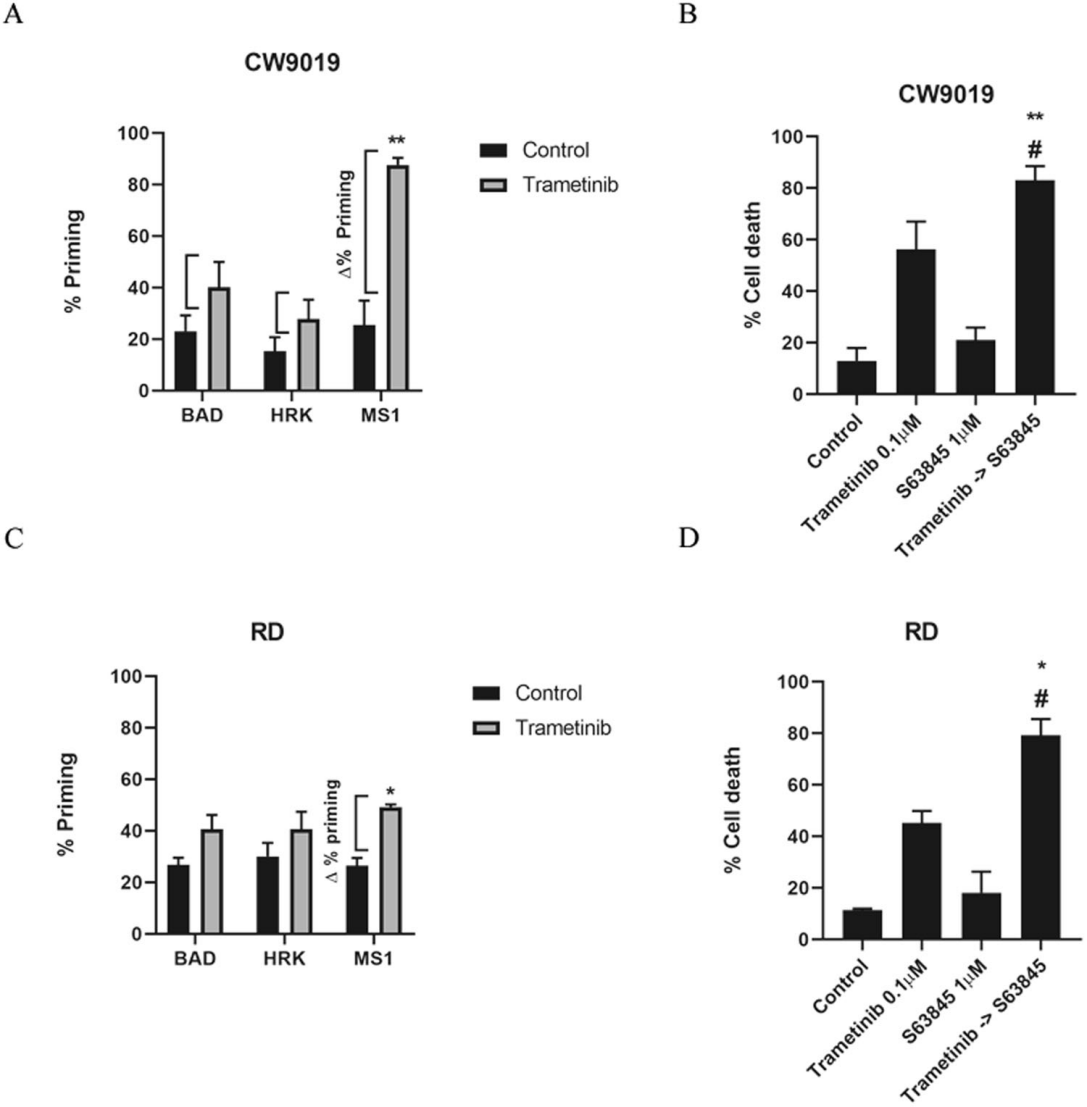
Dynamic BH3 profiling predicts MCL-1 anti-apoptotic adaptation as a resistance mechanism after trametinib treatment in RMS cell lines. **A**, **C** Results from the contribution of each anti-apoptotic protein (BCL-2/BCL-xL dependence BAD peptide, BCL-xL dependence HRK peptide and MCL-1 dependence MS1 peptide) in acquiring resistance to trametinib 0.1 µM treatment in CW9019 and RD respectively. Results expressed as Δ% priming represents the increase in priming compared to control cells. MS1 BH3 peptide showed a significant increase, indicating MCL-1 adaptation after treatment. **B**, **D** Cell death by Annexin V and propidium iodide staining and FACS analysis after 96 h incubation of CW9019 and RD cells with the single agents alone or the combination of trametinib with the BH3 mimetic S63845 for 96 h. Values indicate mean values ± SEM. ** *p* < 0.01, * *p* < 0.05 compared to single agents and # indicates CI < 1. All experiments were performed at least three times.

We focused on deciphering why the three RMS cell lines (CW9019, RD and RH4) [50] responded differently to trametinib as single agent (Fig. 1). Interestingly, we observed that trametinib caused an increase in % priming with the MS1 peptide in all RMS cell lines (Fig. 2A, C and Supplementary Fig. 2A), being the highest increase the one detected in CW9019 cells (Fig. 2A). This indicated that all RMS cells exerted a rapid pro-survival adaptation through the anti-apoptotic protein MCL-1 after trametinib treatment. We then evaluated the sequential treatment of the MCL-1 inhibitor S63845 after trametinib administration in RMS cells. We observed a synergy in both CW9019 cells (Fig. 2B) and RD cells (Fig. 2D) with combination indexes (CI) of 0.79 and 0.69 respectively, indicating that MCL-1 inhibition significantly enhanced trametinib cytotoxic effect. A more modest effect was observed in the ARMS cell line RH4 that exerted an additive effect (CI = 1) (Supplementary Fig. 2B), that could be explained by its lower initial response to the MEK1/2 inhibitor (Supplementary Fig. 1). To further validate this MCL-1 mediated resistance to MEK1/2 inhibition, we tested a second inhibitor, the clinically used selumetinib, in CW9019 cells, obtaining similar results (Supplementary Fig. 3A, B).

### BCL-xL and MCL-1 are key players in RMS cell death resistance

**Fig. 3:**
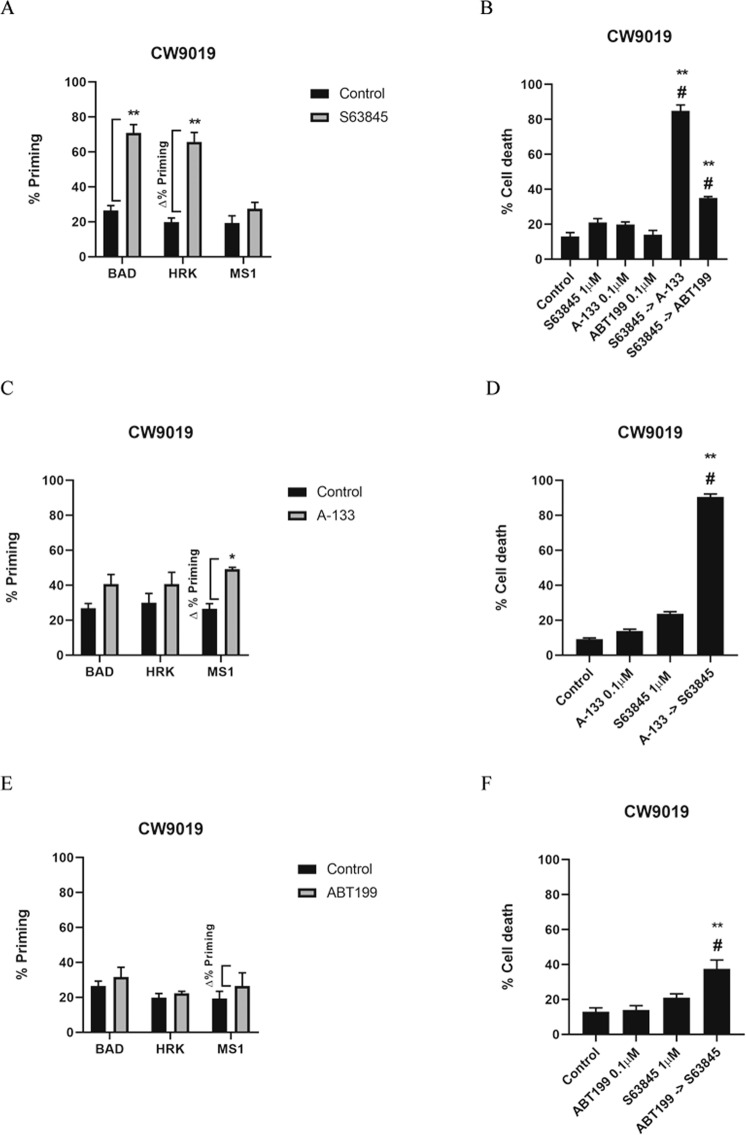
Dynamic BH3 profiling predicts different anti-apoptotic adaptation as a resistance mechanism after BH3 mimetics treatment in CW9019 cell line. **A**, **C**, **E** Results from the contribution of each anti-apoptotic protein (BCL-2/BCL-xL dependence BAD peptide, BCL-xL dependence HRK peptide and MCL-1 dependence MS1 peptide) in acquiring resistance to S63845 1 µM, A-133 0.1 µM and ABT-199 0.1 µM treatment respectively in CW9019 cells. Results expressed as ∆% priming represents the increase in priming compared to control cells. **B**, **D**, **F** Cell death by Annexin V and propidium iodide staining and FACS analysis after 96 h incubation of CW9019 cells with the single agents alone or the combination with the other BH3 mimetics for 96 h. Values indicate mean values ± SEM. ** *p* < 0.01, * *p* < 0.05 compared to single agents and # indicates CI < 1. All experiments were performed at least three times.

**Fig. 4:**
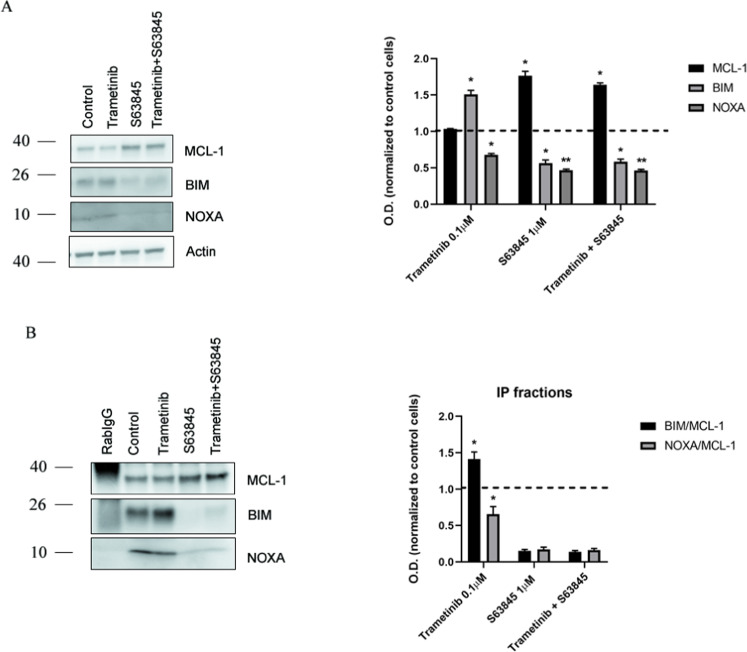
Trametinib causes a decrease in NOXA-MCL-1 binding levels as a resistance mechanism in CW9019 cells. **A** Left panel: Representative images from Western blot analysis of CW9019 cell lysates after the indicated treatments for 16 h (trametinib 0.1 µM, S63845 1 µM) or the sequential combination of both (trametinib 0.1 µM during 16 h followed by S63845 1 µM treatment for 2 h). Right panel: Optical density quantification normalized to actin and represented as fold change compared to control. **B** Western blot results of the co-immunoprecipitation between MCL-1 and BIM/NOXA after 0.1 µM trametinib, 1 µM S63845, or the sequential combination of both treatments. Values indicate mean values ± SEM from at least three independent experiments. **p < 0.01, *p < 0.05. All experiments were performed at least three times.

We then wanted to further study how RMS cells adapt to anti-apoptotic inhibition as previously described in pediatric cancer [39]. Therefore, we sought to pharmacologically inhibit one anti-apoptotic protein using a specific BH3 mimetic and study potential pro-survival adaptations through other BCL-2 family members using DBP. In this regard, CW9019 cells showed a similar increase in % priming with BAD and HRK peptides after MCL-1 inhibition with S63845 (Fig. 3A) indicating a predominant acquired resistance through BCL-xL, but with a possible minor contribution from BCL-2 as well (Fig. 3A). When we performed cell death measurements at 96 h, we could observe that sequential treatment with A-133 after S63845 caused a strong synergy in these cells (CI = 0.43) (Fig. 3B) as predicted by DBP (Fig. 3A), and as previously observed in breast cancer [51]. The sequential addition of ABT-199 to S63845 also improved the cytotoxic effects compared to single agents but to a lower extent (CI = 0.91) (Fig. 3B), in accordance with DBP predictions (Fig. 3A). As MCL-1 appears to be a key protein in RMS progression [20, 21], particularly for CW9019 cells, its inhibition could not be compensated by just one anti-apoptotic protein and requires an increased activity of both BCL-2 and BCL-xL. When CW9019 cells were treated with A-133, we could observe an increase in % priming with the MS1 peptide, indicating an acquired resistance through MCL-1 (Fig. 3C). This prediction was also confirmed by in vitro cell death measurements where we detected a strong synergy when sequentially combining A-133 followed by S63845 (CI = 0.37) (Fig. 3D) as we previously described in another solid tumor [51]. Finally, CW9019 cells showed increased % priming with the MS1 peptide when treated with ABT-199 (Fig. 3E) indicating an acquired resistance through MCL-1 that was further corroborated by the synergistic combination (CI = 0.54) of ABT-199 and S63845 (Fig. 3F), although exerting a modest cytotoxicity. These results showed that BCL-xL and MCL-1 are the most important proteins within the BCL-2 anti-apoptotic members mediating the acquisition of resistance in RMS, validating previous findings by Kehr and colleagues [39], and we believe that BH3 mimetics could be used sequentially to minimize undesired secondary effects. Interestingly, when we analyzed the anti-apoptotic adaptations induced by S63845 as single agent or by trametinib + S63845, in both cases we observed a significant and comparable BCL-xL adaptation as indicated by HRK Δ% priming (Supplementary Fig. 4B, C). Taking into consideration that trametinib, with or without S63845 increased overall BIM Δ% priming (Supplementary Fig. 4A), but as single agent had a minor contribution on BCL-xL adaptation (Fig. 4A), we conclude that S63845 is the main agent inducing this pro-survival change. When we performed cell death analysis comparing all the possible combinations, we observed that trametinib + S63845, the sequential combination of S63845 with A-133 or the sequential combination of trametinib + S63845 followed by A-133 showed similar cytotoxicities (Supplementary Fig. 4D). This data suggests that this triple combination does not improve the efficacy of dual MEK and MCL-1 inhibition, or MCL-1 and BCL-xL co-inhibition.

Finding new targeted agents to specifically engage tumor cells while sparing non-cancer cells to avoid undesired side effects still represents an unmet need for RMS patients. To address this issue, we tested the cytotoxic effect of the targeted therapies previously described in the non-tumoral murine and human myoblast cell lines C2C12 and HSMM. We observed that neither C2C12 cells nor HSMM were affected by single agents, indicating specific toxicity for cancer cells (Supplementary Fig. 5A). Furthermore, we tested in HSMM the most promising combinations previously identified and we could not detect significant cytotoxicity (Supplementary Fig. 5B). In summary, these newly identified therapies against this pediatric disease did not seem to affect non-tumoral cells.

### Trametinib promotes a protective decrease in NOXA: MCL-1 binding

To better understand how RMS cells acquire resistance to trametinib and why its combination with S63845 is synergistic, we analyzed protein expression changes in CW9019. We observed an increased expression of BIM after MEK1/2 inhibition (Fig. 4A), as described elsewhere [52, 53]. As expected, we observed a dramatic decrease in phospho-ERK1/2 in cells treated with trametinib compared to control cells (Supplementary Fig. 6). Surprisingly, we did not observe major changes in the anti-apoptotic proteins BCL-2, BCL-xL, and MCL-1 (Fig. 4A and Supplementary Fig. 6), despite previous in vitro observations (Fig. 2). As reported in the literature, MCL-1 can be blocked by the sensitizer BH3-only protein NOXA, impeding its pro-survival activity and therefore promoting apoptosis [19, 54]. Based on recent studies [46], we hypothesized that this sensitizer protein could explain MCL-1 dependence in RMS cells after trametinib treatment. We immunoprecipitated MCL-1 from drug-treated CW9019 and analyzed its binding to NOXA. After exposing cells to this targeted agent, we could observe a decrease in NOXA binding (Fig. 4B) while MCL-1 expression remained unaltered (Fig. 4A). MCL-1 can also bind to the pro-apoptotic activator protein BIM, so we assessed this interaction and detected an increased binding between these two proteins after MEK1/2 inhibition (Fig. 4B). In brief, fast NOXA downregulation after trametinib exposure, liberates MCL-1 that can then capture more BIM to prevent BAX and BAK activation (and the induction of apoptosis). As previously reported, S63845 promoted the stabilization and accumulation of MCL-1 [55, 56] that could be observed in total cell lysates (Fig. 4A). This BH3 mimetic also caused the displacement of the remaining NOXA bound to MCL-1 and its proteasomal degradation [57], explaining its dramatic reduction in total cell lysates (Fig. 4A). More importantly, the sequential inhibition of MCL-1 after trametinib treatment released BIM and restored apoptosis (Fig. 4B), explaining the observed synergy between this targeted agent and the BH3 mimetic S63845.

### Effective therapeutic combination of trametinib with the MCL-1 inhibitor S63845 in a PDX model of RMS

**Fig. 5:**
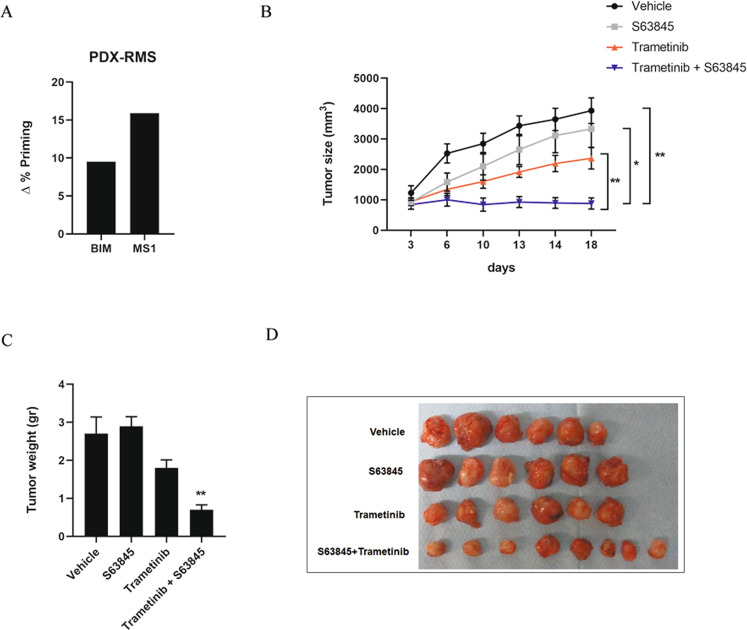
Trametinib and S63845 combination inhibits tumor growth in a PDX model of RMS. **A** Results from the contribution of BIM and MCL-1 dependence MS1 peptide in acquiring resistance to trametinib 0.1 µM in disaggregated cells from a PDX-RMS tumor (*n* = 1). Results expressed as ∆% priming represents the increase in priming compared to control cells. **B** Tumor size (expressed in mm^3^) quantification after treatment with vehicle, S63845, trametinib, and trametinib + S63845. Measurements represent days after initiation of treatment. **C** Tumor weight (expressed in grams) quantification after treating the mice with vehicle, S63845, trametinib, and trametinib + S63845. **D** Image of tumors dissected from PDX mice after treatments. All values indicate mean values ± SEM. ** *p* < 0.01, *n* = 6.

We sought to test this promising therapeutic combination in vivo as trametinib was already assessed in clinical trials for the treatment of pediatric glioma and plexiform neurofibroma [58, 59]. In fact, MEK1/2 inhibition for the treatment of pediatric solid tumors is under evaluation in clinical trials (NCT02285439, NCT02285439), and several MCL-1 inhibitors for adult cancer treatment [60]. We performed DBP analyses on RMS PDX tumor-isolated cells. After disaggregating the cells, we exposed them for 16 h to trametinib, detecting an increase in priming with the BIM peptide (Fig. 5A). In parallel, this same treatment produced a rapid increase in the MS1 signal, indicating MCL-1 adaptation (Fig. 5A), as previously observed in vitro (Fig. 2A, C and Supplementary Figs. 2A, 3A). Thus, DBP can predict both treatment effectiveness and anti-apoptotic adaptations in cell lines and RMS PDX-isolated cells. To further study the effectiveness of the combination of trametinib and S63845 in vivo, we treated PDX mice with the MCL-1 inhibitor as a single agent, or right after trametinib treatment to overcome the detected anti-apoptotic resistance. The sequential combination of both drugs caused a significant reduction of the tumor volume and weight, compared to single agents (Fig. 5B–D). These results demonstrate that DBP can be used to design more effective treatment combinations by overcoming the swift anti-apoptotic resistance acquired right after targeted agents’ treatment to stop cancer progression. We postulate the trametinib and S63845 combination as a potential new treatment for RMS.

## Discussion

The discovery of oncogenes and tumor suppressors was crucial to understand cancer progression [30]. As a result, targeted therapies emerged as novel approaches to treat cancer [61], and many are now used in adult tumors [46, 62], as they often rely on pro-tumorigenic altered kinases that activate key signaling pathways [63]. Notably, the constitutive activation of the mitogen-activated protein kinase (MAPK) signal transduction pathway is the most commonly dysregulated in cancer [63]. In RMS, several receptor tyrosine kinases (RTKs) have been identified as potential druggable targets for RMS including IGF-1R, RAS, ALK, EGFR, and VEGFR [12]. Their dysregulation leads to alterations in signaling pathways such as PI3K/AKT/mTOR, MEK/ERK, and JAK/STAT3 [12]. Specifically, primary samples from RMS patients showed both phosphorylation of ERK1/2 and AKT, indicating an overactivation of these pathways, thus becoming promising druggable targets for this type of pediatric cancer [64]. In this study, we used DBP to identify that MEK1/2 inhibition with trametinib, or selumetinib, and IGR-1R with BMS-754807, prime RMS cells to apoptosis; and we confirmed it using cell death analyses in vitro (Fig. 1 and Supplementary Fig. 1). However, single agent targeted therapies were not particularly effective, mostly because cancer persister cells acquire resistance to them [12]. It has been previously described that a common mechanism to block treatment-induced cell death is upregulation or activation of anti-apoptotic proteins [28, 65]. Therefore, we hypothesized that the combination of targeted agents with BH3 mimetics could be a good strategy to overcome these therapeutic adaptations. However, the vast majority of published preclinical strategies are based on combinations of targeted agents with commonly used chemotherapeutics [12]. Using DBP we could identify MCL-1 anti-apoptotic adaptations [19, 45–48] upon MEK1/2 inhibition in three RMS cell lines and a PDX-RMS model (Figs. 2 and 5A). This was further confirmed by cytotoxicity measurements in vitro, where we observed synergistic combinations between trametinib or selumetinib with the MCL-1 inhibitor S63845 (Fig. 2 and Supplementary Figs. 2 and 3). The effectiveness of this therapeutic sequence was previously reported in different adult cancers [34, 46, 66, 67] but to our knowledge, this is the first time that this rational combination is described for pediatric RMS. Furthermore, we confirmed that these swift anti-apoptotic adaptations occur mostly through MCL-1, in accordance with previous reports [39]; and that BCL-xL and MCL-1 compensate each other when blocked with specific BH3 mimetics. For instance, CW9019 cells acquire resistance to S63845 mainly through BCL-xL in CW9019 (Fig. 3A, B). In contrast, the blockade of BCL-xL with A-133 caused an adaptation through MCL-1 in CW9019 (Fig. 3C, D). The sequential combination of both BH3 mimetics caused a significant increase in cell death in CW9019, indicating that these two proteins are the main regulators of therapy-acquired resistances in CW9019 cells (Fig. 3B, D). The therapeutic potential of simultaneously blocking BCL-xL and MCL-1 has been reported in many cancers [39, 68, 69], and we recently demonstrated this strategy in ER + breast cancer using a sequential regime [51], although BCL-xL induced thrombocytopenia may challenge its clinical implementation. In addition, we identified that the sequential combination of ABT-199 and S63845 was more effective in CW9019 cells than single agents and was also predicted by DBP (Fig. 3E, F). This effective co-inhibition of BCL-2 and MCL-1 using BH3 mimetics was previously reported in hematological malignancies [70] but not RMS. Overall, most reports describe simultaneous co-inhibition of anti-apoptotic proteins, but according to our data, a sequential combination of treatments would be more effective and potentially decrease secondary effects [19]. Surprisingly, the triple inhibition of MEK, MCL-1, and BCL-xL was not more cytotoxic than the described double inhibitions (Supplementary Fig. 4).

**Fig. 6:**
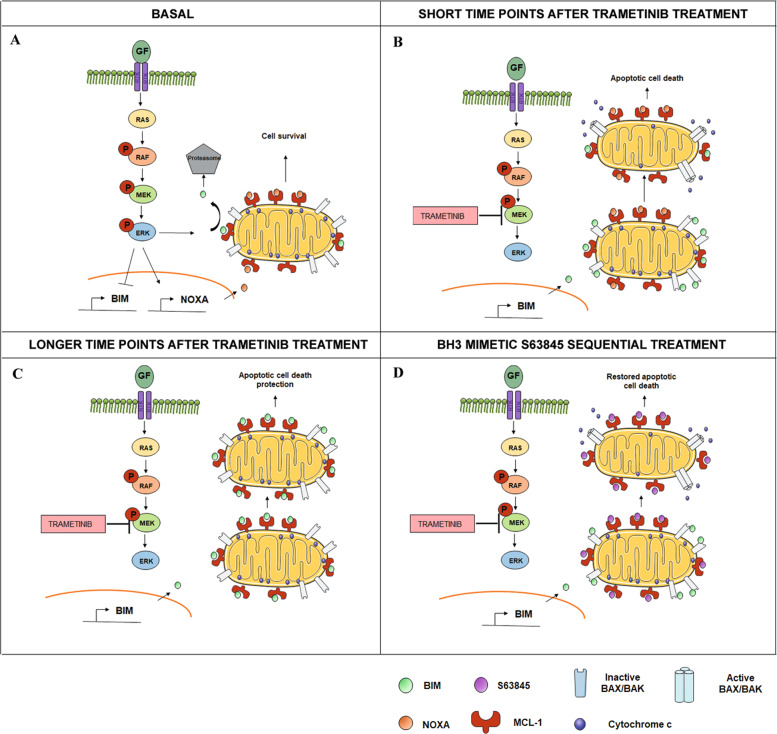
Use of S63845 to overcome RMS cells’ resistance to trametinib. **A** Schematic representation of the basal situation in CW9019 cells. **B** When CW9019 cells are exposed to trametinib there is a decrease in NOXA transcription, which leads to an increase in the availability of MCL-1. **C** After longer incubations with trametinib, BIM is sequestered by MCL-1 promoting apoptotic cell death protection. **D** Apoptosis is restored by the sequential addition of the BH3 mimetic S63845.

From all the identified treatments, we focused on trametinib based on the efficacy of MEK1/2 inhibition in RMS preclinical studies [71], and prior promising clinical studies in pediatric glioma patients with active MAPK signaling [58, 59]. We aimed to elucidate the molecular mechanism driving the rapid MCL-1 acquired-resistance to trametinib in RMS [50]. We focused on CW9019 because it is an ARMS cell line, the RMS subtype with lower prognosis [6, 50], and displayed the highest increase in % priming with the MS1 peptide after trametinib exposure (Fig. 2A). We observed an upregulation of BIM after trametinib treatment in CW9019 cells (Fig. 4A), as previously reported [52, 53, 72–74], explaining the observed cell death increase in RMS cells (Fig. 1B–D). However, MEK1/2 inhibition with trametinib did not produce any change in the anti-apoptotic protein levels in RMS cells (Fig. 4A and Supplementary Fig. 6). The MCL-1/NOXA axis regulates apoptosis [46, 75–78], since the sensitizer NOXA can block MCL-1 and indirectly promote apoptosis [19, 54]. We observed a significant decrease in NOXA binding to MCL-1 after trametinib treatment in CW9019 cells (Fig. 4B), thus increasing the availability of this anti-apoptotic protein to avoid therapy-induced cell death. Indeed, we could observe an increment in BIM and MCL-1 binding after trametinib exposure, preventing BAX/BAK activation and the induction of apoptosis (Fig. 2). In brief, when RMS cells are exposed to trametinib, ERK1/2 is dephosphorylated and its downstream signaling repressed, leading to a decrease in NOXA expression (Fig. 6B) and BIM proteasomal degradation that restores its transcription [72]; allowing MCL-1 to sequester BIM and protect cells from apoptosis (Fig. 6C). The sequential addition of the BH3 mimetic S63845 to trametinib, displaces BIM from MCL-1, promoting BAX/BAK activation and cell death restoration (Fig. 6D). We also explored if this resistance mechanism and the therapeutic combination that we found in RMS cell lines could be also efficient in vivo using a PDX model of RMS. First, we detected an increase in Δ% priming with MS1 after trametinib treatment (Fig. 5A) in cells isolated from a RMS-PDX tumor, which correlated with the in vitro findings (Fig. 2). Furthermore, when we treated RMS-PDX mice with this therapeutic combination we found a significant reduction of the tumor size and volume compared to vehicle and single agents (Fig. 5B–D).

In conclusion, this work demonstrates how the functional assay DBP can predict targeted agents’ anti-tumor efficacy and anti-apoptotic adaptations in RMS. These adaptations occur rapidly, in less than 24 h, but may explain why some of these agents often fail to completely eliminate cancer cells. Sequential combinations of targeted agents with BH3 mimetics can greatly improve RMS treatment while decreasing potential secondary effects in the clinic. We postulate trametinib as a novel effective targeted agent to treat RMS, similarly as in pediatric glioma [58, 59], when metronomically combined with MCL-1 inhibitors such as S63845; also in ARMS, a RMS subtype that has a high propensity for metastases and presents poor prognosis [6, 50], may be clinically relevant. Our results suggest that this new therapeutic strategy should be tested in RMS patients, to avoid cancer persister cells’ survival and relapse.

## Materials and methods

### Cell lines and treatments

RMS cell lines (CW9019, RD and RH4) were kindly provided by Dr. Oscar Martínez-Tirado and Dr. Cristina Muñoz-Pinedo from the Biomedical Research Institute from Bellvitge (IDIBELL). C2C12 cells and Human skeletal muscle myoblasts (HSMM) were purchased at ATCC (ATCC^®^ CRL-1772™, ATCC, Manassas, Virginia, USA) and Lonza (CC-2580, Lonza, Basel, Switzerland), respectively. RMS cell lines were maintained in RPMI 1640 medium (31870, Thermo Fisher, Gibco, Paisley, Scotland) and supplemented with 1% of L-Glutamine (25030, Thermo Fisher, Gibco) and 1% of penicillin and streptomycin (15140, Thermo Fisher, Gibco) and 10% heat-inactivated fetal bovine serum (10270, Thermo Fisher, Gibco). C2C12 cells were cultured in DMEM high glucose medium (41965, Thermo Fisher, Gibco) supplemented with 10% heat-inactivated fetal bovine serum and 1% of penicillin and streptomycin. HSMM cells were maintained in SKBM-2 medium (CC-3246, Lonza) supplemented with its specific SingleQuots^TM^ and growth factors (CC-3244, Lonza). All cells were kept at 37 °C in a humidified atmosphere of 5% CO_2_. In addition, all of them were routinely tested for mycoplasma. Drug treatments were carried out directly in the culture media. Doses and time points are indicated in every single experiment. All drugs were purchased at Selleckchem (Munich, Germany).

### Dynamic BH3 profiling

Dynamic BH3 profiling was performed as previously described [45, 46]. Briefly, 3 × 10^5^ cells were incubated with targeted therapies (or DMSO in the control condition) for 16 h at 37 °C. Afterwards, cells were stained with the viability marker Zombie Violet (423113, BioLegend, Koblenz, Germany) for 10 min at room temperature (R.T.) and then washed with PBS and resuspended in 330 µl of MEB (150 mM mannitol, 10 mM hepes-KOH pH 7.5, 150 mM KCl, 1 mM EGTA, 1 mM EDTA, 0.1% BSA, 5 mM succinate). Simultaneously, 12 different peptide solutions were prepared in MEB with 0.002% digitonin (D141, Sigma-Aldrich). The final concentration of each peptide solution was: 10, 3, 1, 0.3, 0.1, 0.03, and 0.01 µM of BIM BH3 peptide, 10 µM of BAD BH3 peptide, 100 µM of HRK BH3 peptide, 10 µM of MS1 BH3 peptide, 25 µM of alamethicin (BML-A 150-0005, Enzo Life Sciences, Lörrach, Germany) and DMSO in the control condition. Subsequently, 25 µl of cell suspensions were incubated with 25 µl of each peptide solution in a 96-well plate (3795, Corning, Madrid, Spain) for 1 h at R.T., followed by fixation with formaldehyde and further staining with cytochrome c antibody (Alexa Fluor^®^ 647—6H2.B4, 612310, BioLegend). Individual DBP analysis were performed in triplicates for DMSO, alamethicin, multiple BIM BH3 concentrations, BAD, HRK, and MS1 BH3 peptides. The different analyses were performed with a high-throughput flow cytometry SONY instrument (SONY SA3800, Surrey, United Kingdom). % priming represents the maximum % cytochrome c released obtained after BH3 peptide exposure and Δ% priming stands for the maximum difference between treated cells vs non-treated cells.

### Cell death analysis

After 96 h of incubation with the specified treatments, cells were analyzed using Annexin V (FITC Annexin V, 640906, BioLegend) and propidium iodide (PI) (1056, BioVision, Milpitas, California, USA) and analyzed on a flow cytometry Gallios instrument (Beckman Coulter, Nyon, Switzerland). We considered viable cells when both Annexin V and PI were negative. Results were represented as the mean of % cell death (100 - % viable cells) of at least three independent replicates.

### Protein extraction and quantification

RIPA buffer (150 mM NaCl, 5 mM EDTA, 50 mM Tris-HCl pH = 8, 1% Triton X-100, 0.1% SDS, EDTA-free Protease Inhibitor Cocktail (4693159001 Roche, Mannkin, Germany)) was used to extract proteins from cells as described elsewhere [28]. After 30 min incubation on ice, suspensions were centrifuged at 4 °C for 10 min at 16 100 × *g* and the supernatant was collected and stored at −20 °C. Protein quantification was performed using Pierce^TM^ BCA Protein Assay Kit (23227, Thermo Fisher).

### Immunoprecipitation

The immunoprecipitation protocol used was described previously [28]. Briefly, the immunoprecipitation buffer (150 mM NaCl, 10 mM Hepes, 2 mM EDTA, 1% Triton, 1.5 mM MgCl2, 10% glycerol, EDTA-free Protease Inhibitor Cocktail (4693159001 Roche), PhosSTOP^TM^ (4906845001 Roche)) was used to lysate the cells. Cells were then centrifuged and supernatants were incubated at 4 °C overnight with magnetic beads (161–4021, Bio-Rad, Madrid, Spain) previously conjugated to 5 μg of rabbit anti-MCL-1 antibody (CST94296, Cell Signaling, Leiden, The Netherlands) or 5 μg of rabbit IgG antibody (CST2729, Cell Signaling). After magnetization, supernatant was discarded and the binding fraction was resuspended in 40 μL 4X SDS-PAGE sample buffer and heated at 70 °C for 10 min. Finally, the sample was magnetized to collect the supernatant, which was stored at −80 °C for further analysis.

### Immunoblotting

Proteins were separated and detected as previously described [28]. In brief, SDS-PAGE gel (Mini-Protean TGX Precast Gel 12%, 456–1045, Bio-Rad) was used to separate proteins and then transferred to PVDF membranes (10600023, Amersham Hybond, Pittsburgh, PA, USA). Blocking of membranes was achieved by using 5% dry milk dissolved in Tris Buffer Saline with 1% Tween 20 (TBST) and the following antibodies were incubated overnight at 4 °C: rabbit anti-BCL-2 (CST4223, Cell Signaling), rabbit anti-BCL-xL (CST2764, Cell Signaling), rabbit anti-MCL-1 (CST5453, Cell Signaling), rabbit anti-NOXA (CST14766, Cell Signaling), rabbit anti-BIM (CST2933, Cell Signaling), rabbit anti-phospho-ERK1/2 (CST4376, Cell Signaling), rabbit anti-Actin (CST4970, Cell Signaling). Anti-rabbit IgG HRP-linked secondary antibody (CST7074, Cell Signaling) was used and immunoblots were developed using Clarity ECL Western substrate (1705060, Bio-Rad). When required, immunoblots were stripped in 0.1 M glycine pH 2,5, 2% SDS for 40 min and washed in TBS. The visualization of the bands was done using the LAS4000 imager (GE Healthcare Bio-Sciences AB, Uppsala, Sweden) and ImageJ was then used to quantify the integrated optical density of bands.

### Animals and human tissue

For this study, we used six-week-old female athymic nu/nu mice (Envigo) weighing 18–22 g. These animals were kept in sterile conditions, with autoclaved cages, bedding, water, and food, with 12 h of light and 12 h of dark cycle. Because of the patients’ young age, their tutors gave written consent for research purposes. The in vivo design of experiments was approved by the IDIBELL animal facility committee (AAALAC Unit1155) and the Institutional Ethics Committees approved the study protocol. All experiments followed the Ethical Conduct in the Care and Use of Animals guideline as indicated by The International Guiding Principles for Biomedical Research Involving Animals, developed by the Council for International Organizations of Medical Sciences.

### Development of rhabdomyosarcoma orthoxenograft mouse model

The embryonal rhabdomyosarcoma (ERMS) orthoxenograft/PDOX was generated from a breast metastasis taken at a relapsed time after post-chemotherapy and radiotherapy treatments from a 14-year-old girl. Primary tumor growth was in the perianal region and was metastatic at diagnosis time having both lymph node and bone metastases. The patient’s tutors gave written consent to participate in the study. Under isoflurane anesthesia, the tumor was implanted into the breast and into the right leg. Briefly, for breast implantation, a small fragment (4–6 × 4–6 mm^3^) was fixed with non-absorbable polypropylene suture (Prolene 7.0) into the mammary fat pad, while for leg implantation it was fixed to the muscle of the upper thigh of the right leg. After implantation, tumor formation was checked every week. Orthotopic tumor (named RMSX2) became apparent 1–2 months after engraftment in both locations. When orthotopic tumors reached a volume of around 1500 mm^3^, the animals were sacrificed, and tumors were expanded to three different animals in order to perpetuate the tumor for drug experiments. To perform later analyses, tumors were paraffin-embedded, frozen, and cryopreserved in 10% DMSO + 90% non-inactivated fetal bovine serum (10270, Thermo Fisher, Gibco) to ensure viability.

### Drug treatment in ERMS RMSX2 orthoxenograft tumor

A mouse harboring RMSX2 tumor orthotopically growing in the upper thigh of the right leg (at passage#3) was sacrificed, and tumors were collected and divided into small pieces of approximately 4 × 4 mm^3^. These fragments were then grafted in 30 young female mice. Once tumoral masses reached a similar size of 1000–1200 mm^3^, 28 of these mice were randomly assigned to distinct treatment groups (*n* = 6 to 8/group): (i) Placebo; (ii) Trametinib (1 mg/kg); (iii) S63845 (20 mg/kg); and (iv) combined Trametinib (1 mg/kg) plus S63845 (20 mg/kg). S63845 treatment was applied by tail vein injection (i.v) during three consecutive days per week while trametinib was oral gavage (p.o) during five consecutive days per week) and treatments carried out over a period of three weeks. Trametinib was diluted in 10% cremophor EL/10% PEG400, while S63845 it was in 10% DMSO/40% PEG 300/5% Tween 80/saline. Tumors were measured every 1–4 days using a caliper and their volume was calculated as *v* = (*w*² × *l*)/2, where l stands as the longest diameter and w the width. After sacrifice, tumors were examined and weighted. After dissection, the tissue samples were fixed and for paraffin embedding or viably frozen for later experiments.

### PDX cell isolation

Isolation of single cells from primary tumors from PDX animals was performed as described previously [28]. Briefly, tumors were processed mechanically using the gentleMACS Dissociator (Miltenyl Biotec, Madrid, Spain), and then exposed to an enzymatic digestion solution composed by 125 units of DNAse I (DN25, Sigma-Aldrich, Buchs, Switzerland), 100 units of Hyaluronidase (H3506, Sigma-Aldrich) and 300 units of collagenase IV (17104–019, Thermo Fisher, Gibco) for two rounds of 30 min at 37 °C in constant agitation. The suspension was filtered using a 70-micron filter, red blood cells were eliminated by doing an osmotic shock (100 μL of ice cold water were added for 15 s and then diluted to 50 mL with PBS) and cells were finally resuspended in RPMI medium, seeded 3 × 10^5^ cells/well in a 12-well plate, and treated with the drugs of interest. Dynamic BH3 profiling analysis were performed after 16 h of incubation with the specified drugs.

### Statistical analysis

Statistical significance of the results was carried out using Student’s t-tail test, considering significant *p < 0.05 and **p < 0.01. SEM stands for Standard Error of the Mean. For ROC curve analysis, the threshold between responders and non-responders was considered to be Δ% cell death > 20%. Drug synergies were established based on the Bliss Independent model as previously described [79], where Combinatorial index (CI) was calculated CI = ((D_A_ + D_B_) − (D_A_*D_B_))/D_AB_ (D represents cell death of compound A or B or the combination of both). CI < 1 stands for the presence of synergy in the combination of drugs. The statistical analysis and graph generation were performed using GraphPad Prism8.

## Supplementary information

Supplementary information

Supplemental material

## Data Availability

The corresponding author will provide the original data used to support the findings of this study upon reasonable request.
