# Supplementary material for: MEK and MCL-1 sequential inhibition synergize to enhance rhabdomyosarcoma treatment
Source: Cell Death Discov. 2022 Apr 7;8:172. doi: 10.1038/s41420-022-00959-w (PMC8989976; doi:10.1038/s41420-022-00959-w)
Supplement: Supplementary file 1 — Supplementary information [file 41420_2022_959_MOESM1_ESM.pdf]

Supplementary Figure 1

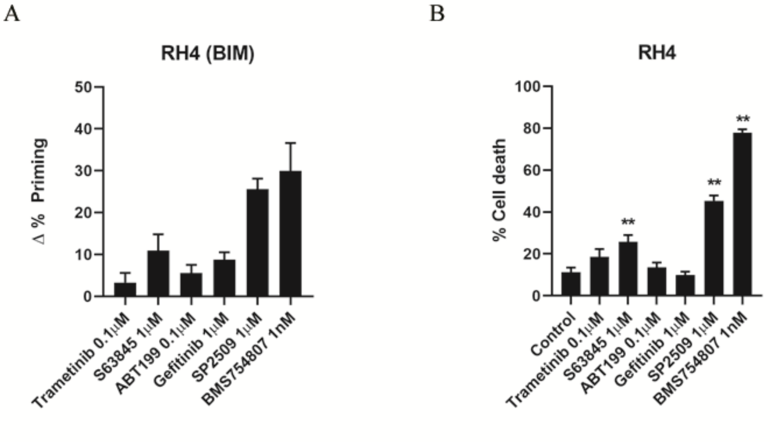

Supplementary Figure 1: Dynamic BH3 profiling predicts sensitivity to targeted agents in RH4 cells. *(A) Results from the DBP assay after 16 hours incubation with treatments in RH4 cells. Results expressed as Δ% priming represents the increase in priming compared to control cells. (B) Cell death results from Annexin V and propidium iodide staining and FACS analysis after 96 hours incubation with the targeted agents in RH4 cells. Values indicate mean values ± SEM from at least three independent experiments. **p<0.01, *p<0.05. All experiments were performed at least three times.*

Supplementary Figure 2

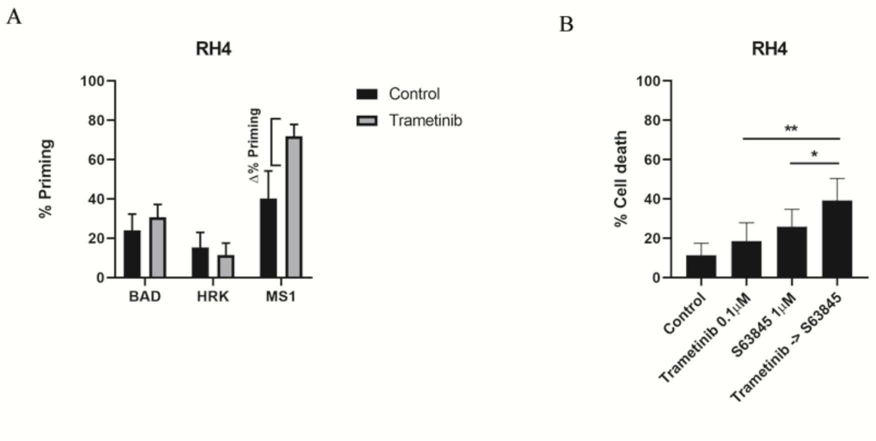

Supplementary Figure 2: Dynamic BH3 profiling predicts MCL-1 anti-apoptotic adaptation as a resistance mechanism after trametinib treatment in RH4 cells. *(A) Results from the contribution of each anti-apoptotic protein: BCL-2/BCL-xL dependence BAD peptide; BCL-xL dependence HRK*

*peptide; and MCL-1 dependence MS1 peptide in acquiring resistance to trametinib 0.1µM treatment in RH4 cells. Results expressed as ∆% priming represents the increase in priming compared to control cells. MS1 BH3 peptide showed a significant increase, indicating MCL-1 adaptation after treatment. (B) Cell death from Annexin V and propidium iodide staining and FACS analysis after 96 hours incubation of RH4 cells with the single agents alone or the combination of trametinib with the BH3 mimetic S63845 for 96 hours. Values indicate mean values ± SEM. ** p< 0.01, * p<0.05 compared to single agents. All experiments were performed at least three times.*

Supplementary Figure 3

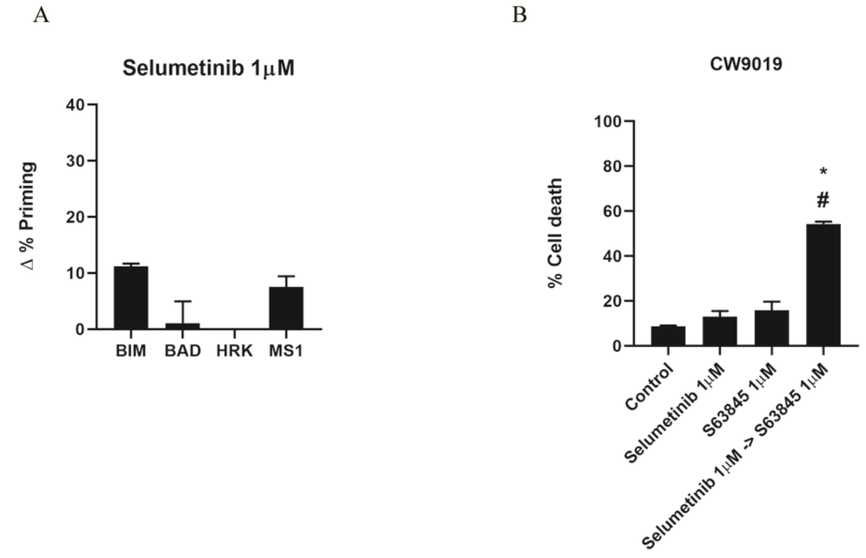

Supplementary Figure 3: CW9019 cells acquire resistance to selumetinib through MCL-1. *(A) DBP results with BIM, BAD, HRK and MS1 after 16 hours treatment with 1µM selumetinib. MS1 BH3 peptide showed a significant increase, indicating MCL-1 adaptation after treatment. (B) Cell death results from Annexin V and propidium iodide staining and FACS analysis after 96 hours incubation with selumetinib 1µM, S63845 1µM or the sequential combination of both. Values indicate mean values ± SEM. * p<0.05 compared to single agents and # indicates CI<1. All experiments were performed at least three times.*

Supplementary Figure 4

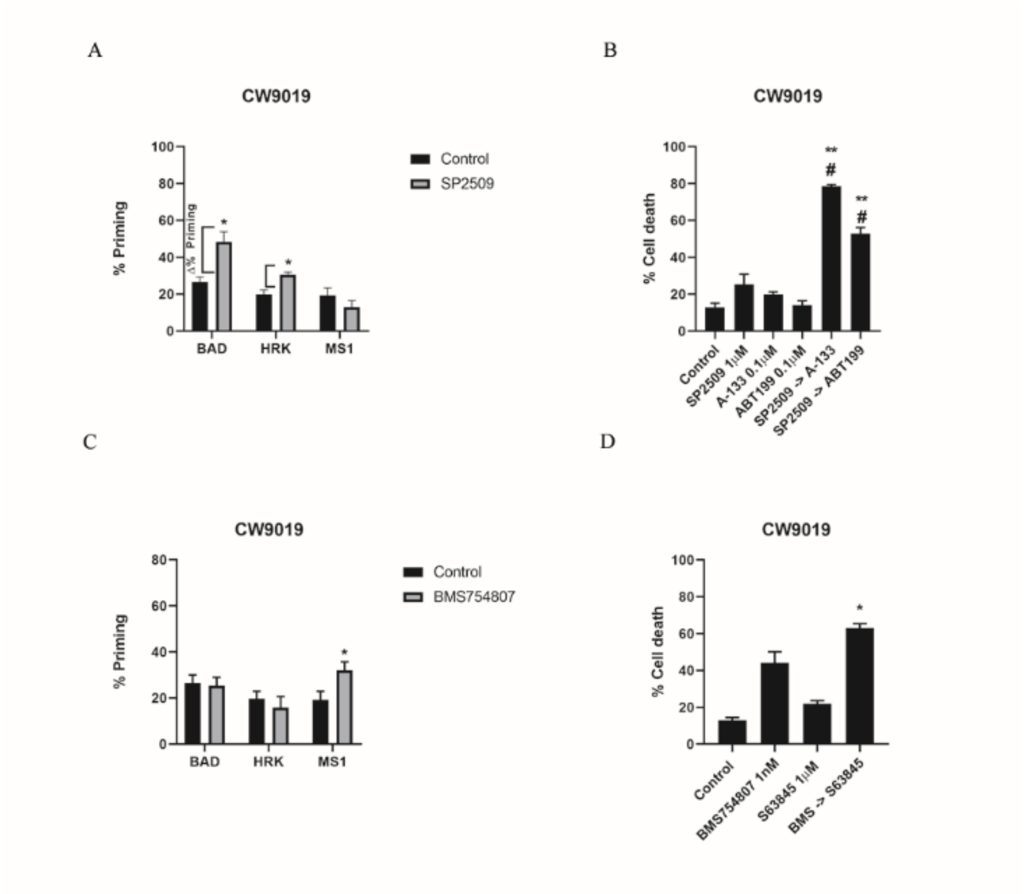

Supplementary Figure 4: MCL-1 inhibition is the main inducer of BCL-xL adaptation. *(A) DBP results with BIM, after 16 hours treatment with 0.1µM trametinib, 1µM S63845 and 0.1µM trametinib + 1µM S63845. No significant Δ% priming changes were observed between trametinib and trametinib + S63845. (B) DBP results with BAD, HRK and MS1 after 16 hours treatment with 1µM S63845. HRK BH3 peptide showed a significant increase, indicating BCL-xL adaptation after treatment. (C) DBP results with BAD, HRK and MS1 after 16 hours treatment with 0.1µM trametinib + 1µM S63845. HRK BH3 peptide showed a significant increase, indicating BCL-xL adaptation after treatment. (D) Cell death results from Annexin V and propidium iodide staining and FACS analysis after 96 hours incubation with indicated agents or combinations. No significant differences were found between trametinib + S63845, the sequential combination of S63845 with A-133 or the sequential combination of trametinib + S63845 followed by A-133. Values indicate mean values ± SEM. **p<0.01 compared to control, ns stands for non-significant. All experiments were performed at least three times.*

Supplementary Figure 5

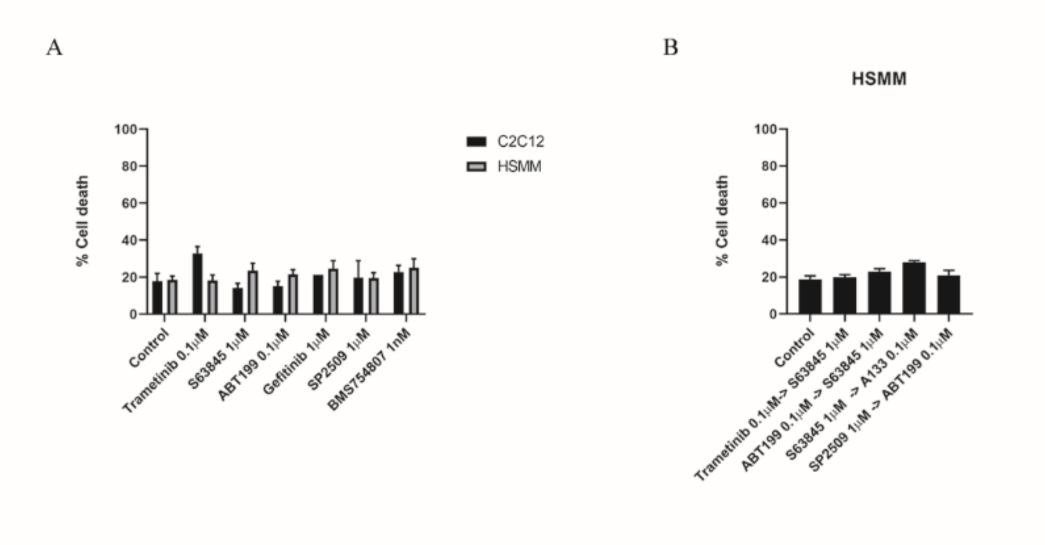

Supplementary Figure 5: Targeted agents and their combination with BH3 mimetics have no effect in non-tumoral cells. *(A) Cell death results from Annexin V and propidium iodide staining and FACS analysis after 96 hours incubation of C2C12 and HSMM with the targeted agents for 96 hours. (B) Cell death results from Annexin V and propidium iodide staining after treating HSMM 96 hours with the most effective combinations of treatments selected in RMS cells. Values indicate mean values ± SEM from at least three independent experiments.*

Supplementary Figure 6

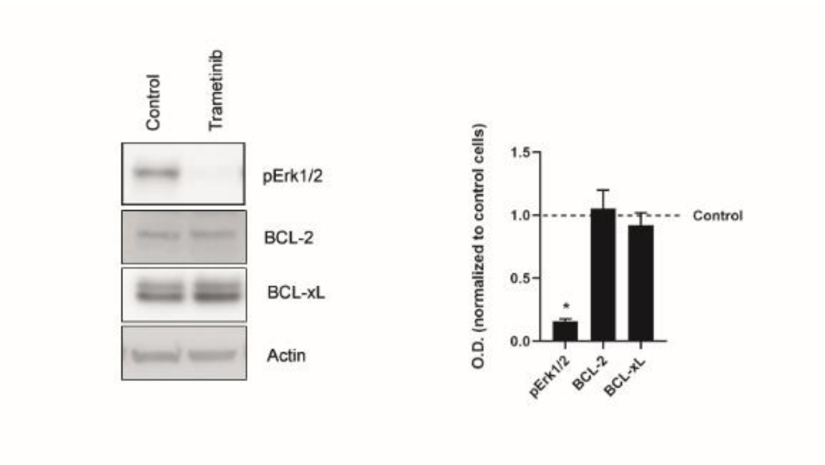

Supplementary Figure 6: Trametinib treatment does not affect the expression of anti-apoptotic BCL-2 proteins in CW9019 cells. *Left panel: Representative images from Western blot analysis of ERK1/2*

*phosphorylation, BCL-2 and BCL-xL in CW9019 cell lysates after trametinib 0.1µM treatment for 16 hours. Right panel: Optical density quantification normalized to actin and represented as fold change compared to control. Values indicate mean values ± SEM. * $p<0.05$. All experiments were performed at least three times.*
