# Supplementary material for: MEK and MCL-1 sequential inhibition synergize to enhance rhabdomyosarcoma treatment
Source: Cell Death Discov. 2022 Apr 7;8:172. doi: 10.1038/s41420-022-00959-w (PMC8989976; doi:10.1038/s41420-022-00959-w)
Supplement: Supplementary file 2 — Supplemental material [file 41420_2022_959_MOESM2_ESM.pdf]

Supplemental material: Western blot images

Figure 4A

Inputs and immunoprecipitated fractions were run together in the same membrane. In figure 4A we show the inputs (left part of the image).

MCL-1

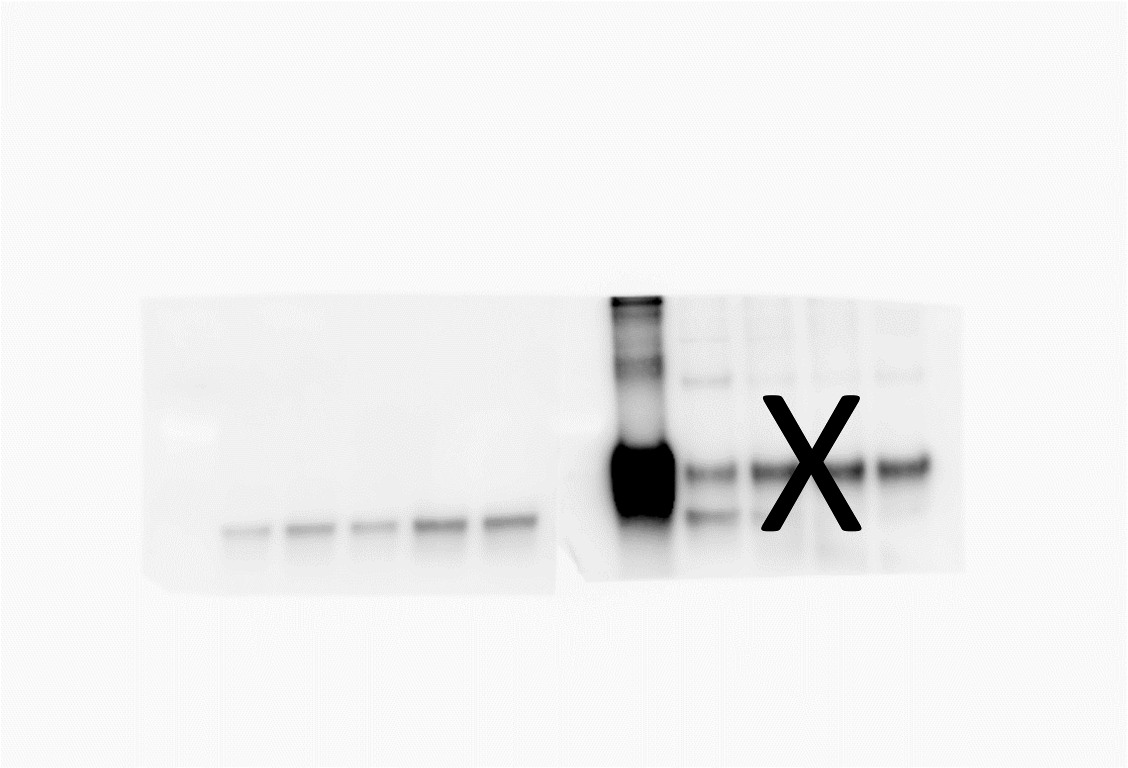

BIM

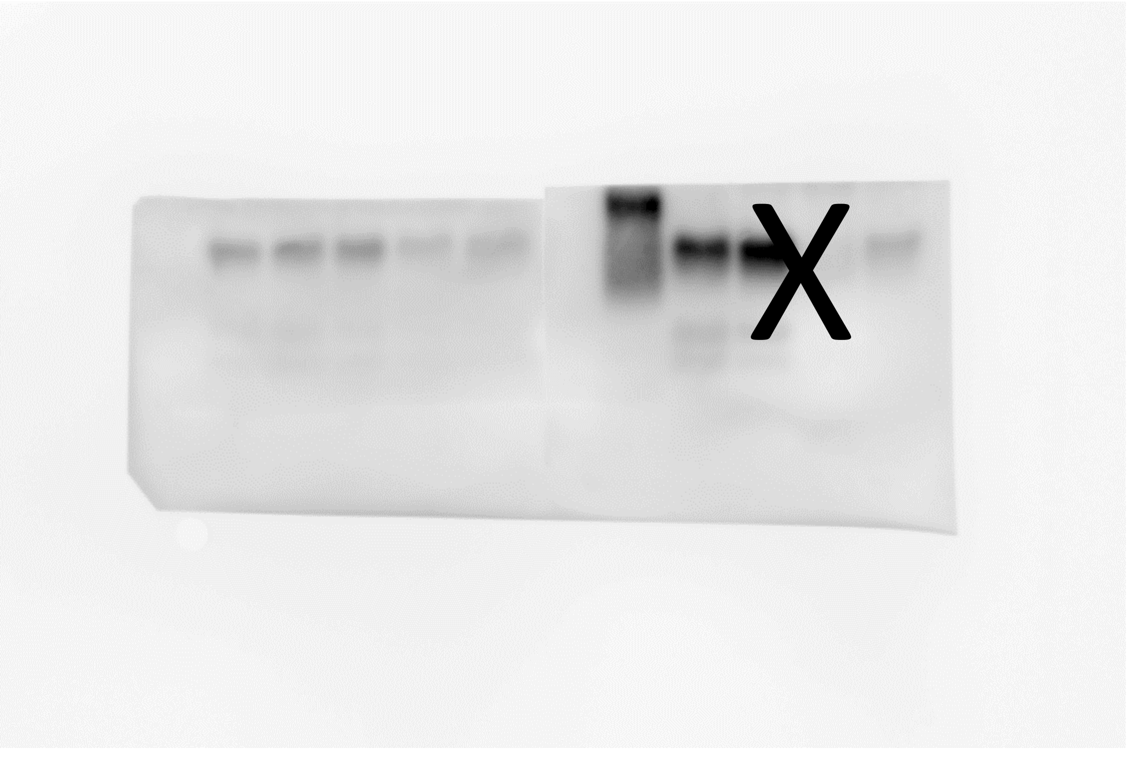

NOXA

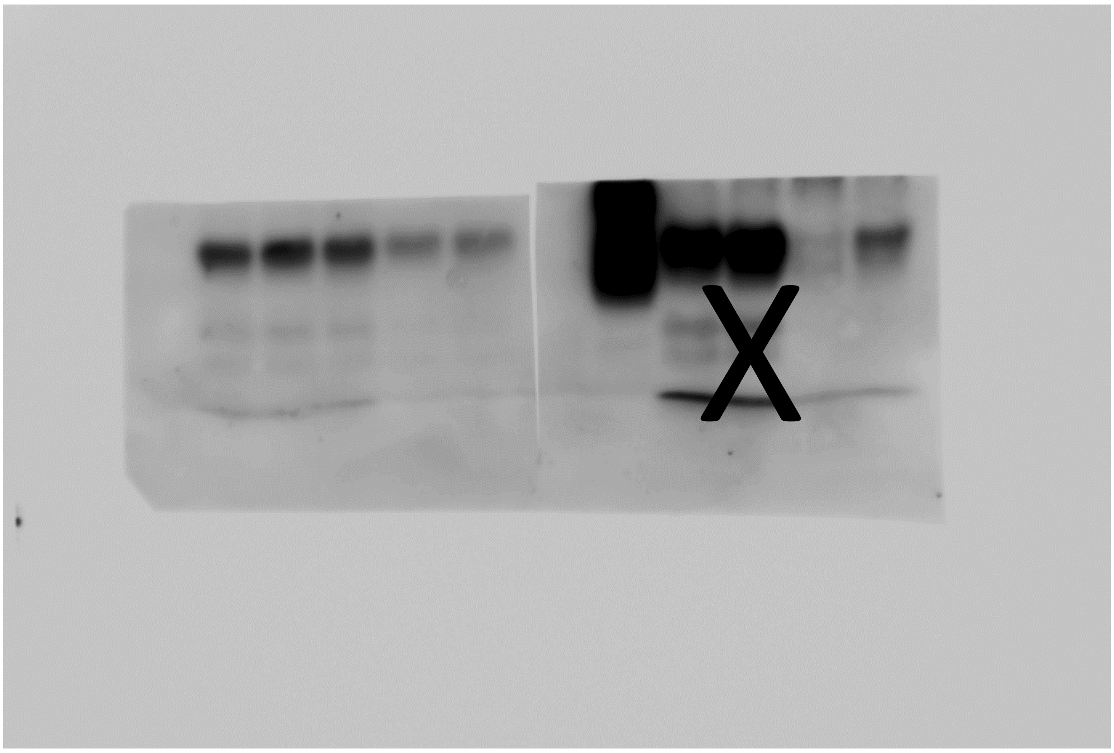

Actin

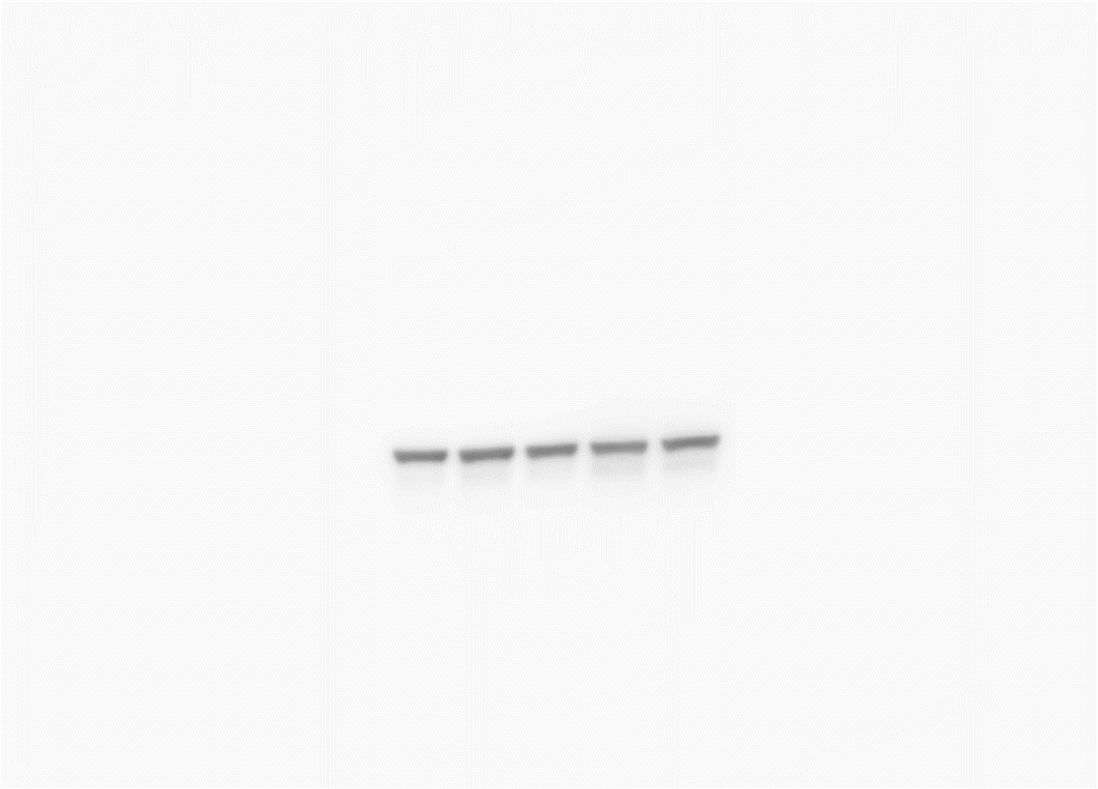

Figure 4B

Inputs and immunoprecipitated fractions were run together in the same membrane. In figure 4B we show the immunoprecipitated fractions (right part of the image).

MCL-1

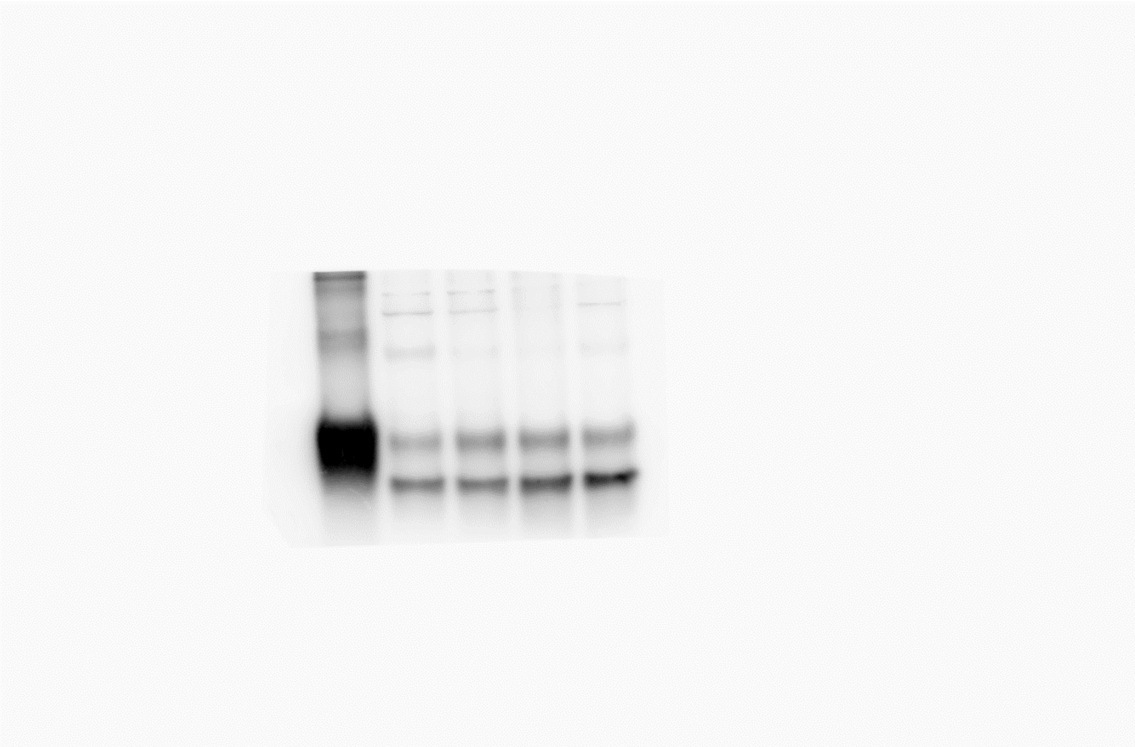

BIM

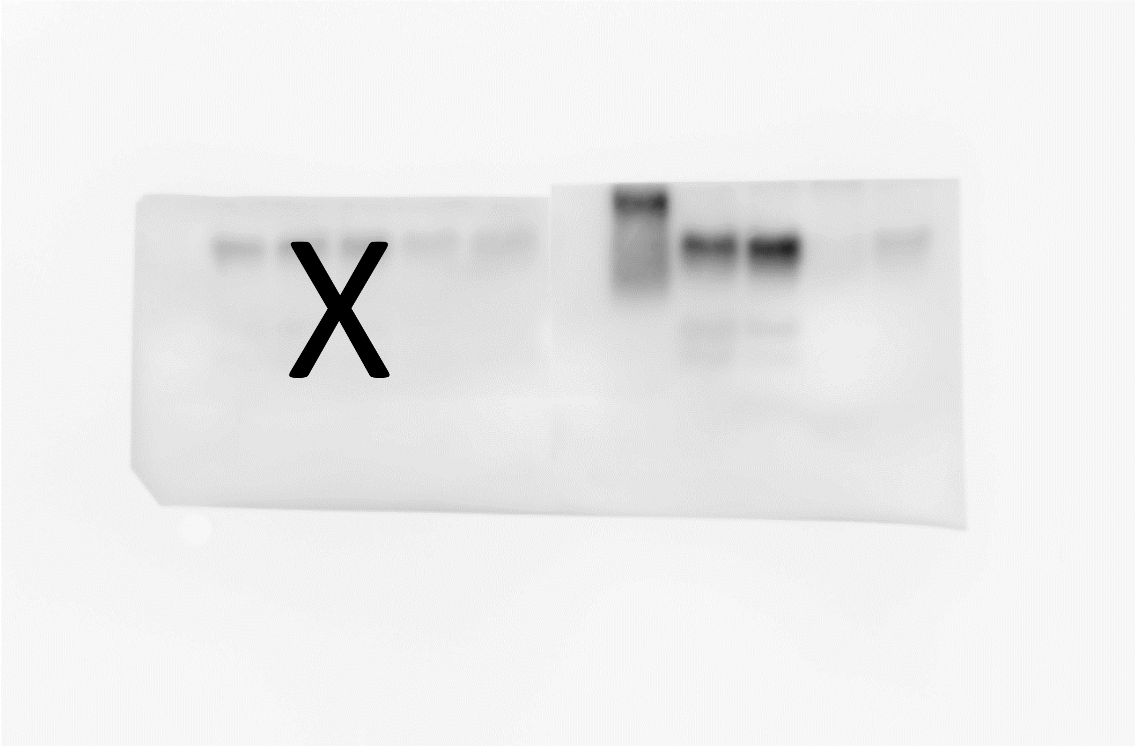

NOXA

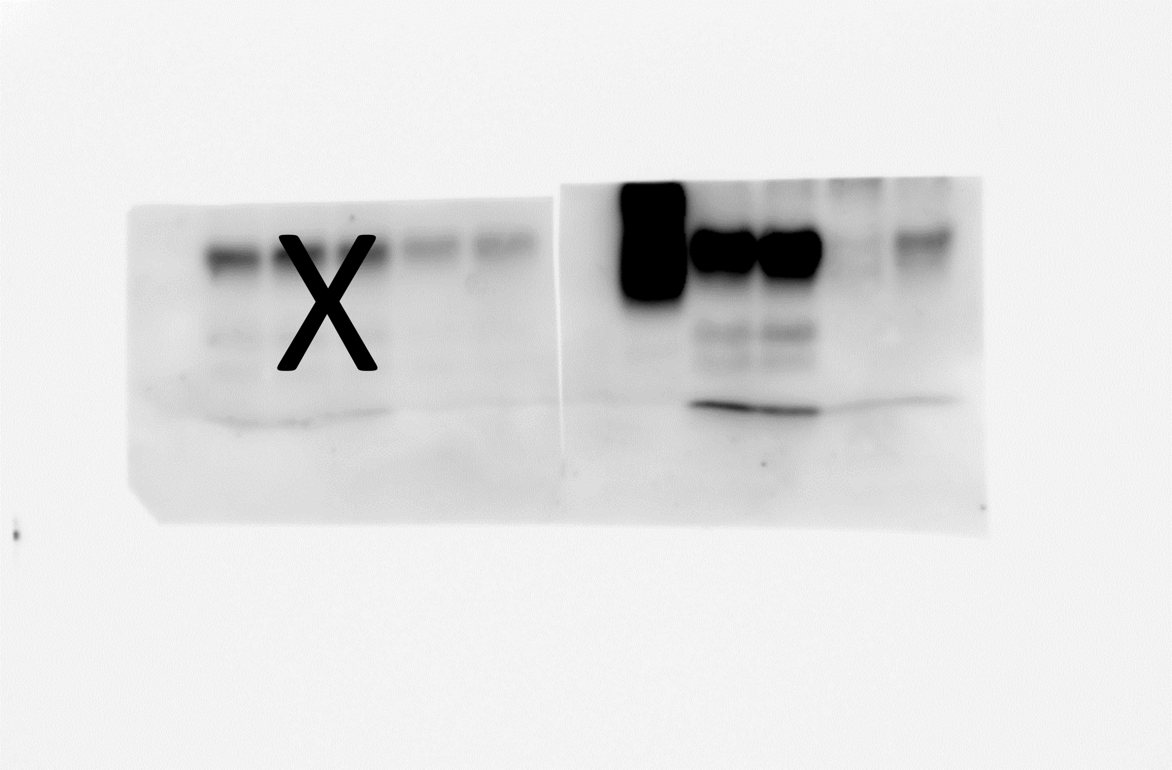

Supplementary Figure 6

pErk

BCL-xL

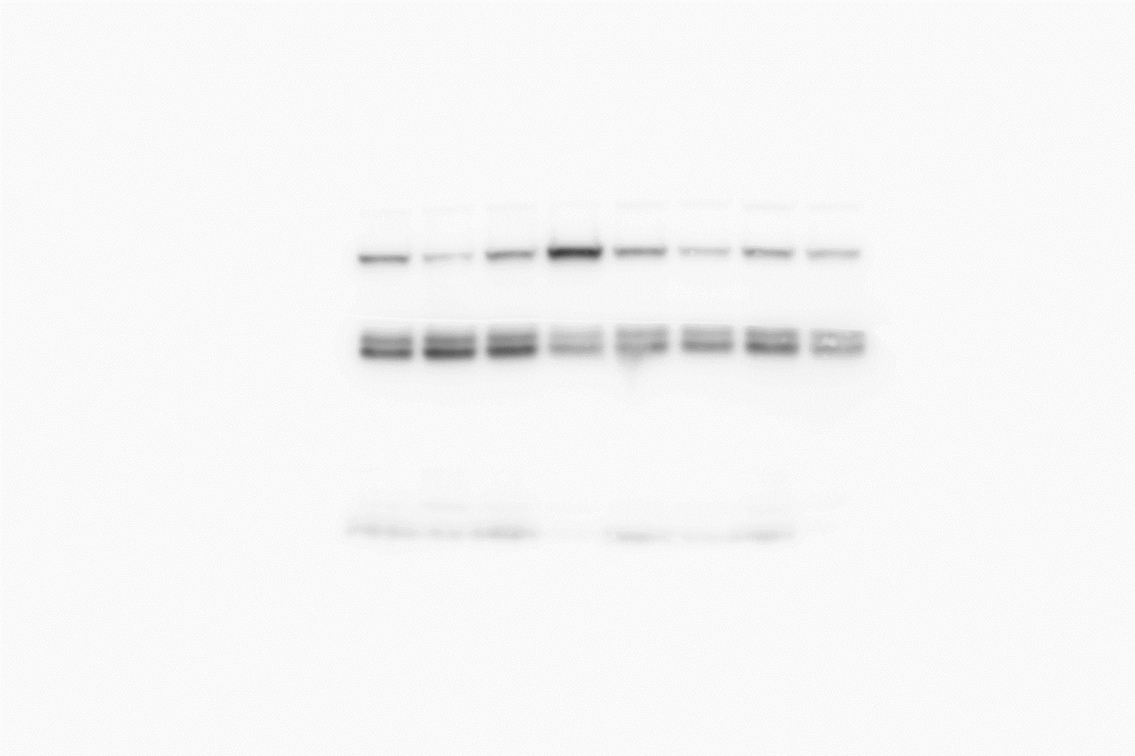

BCL-2

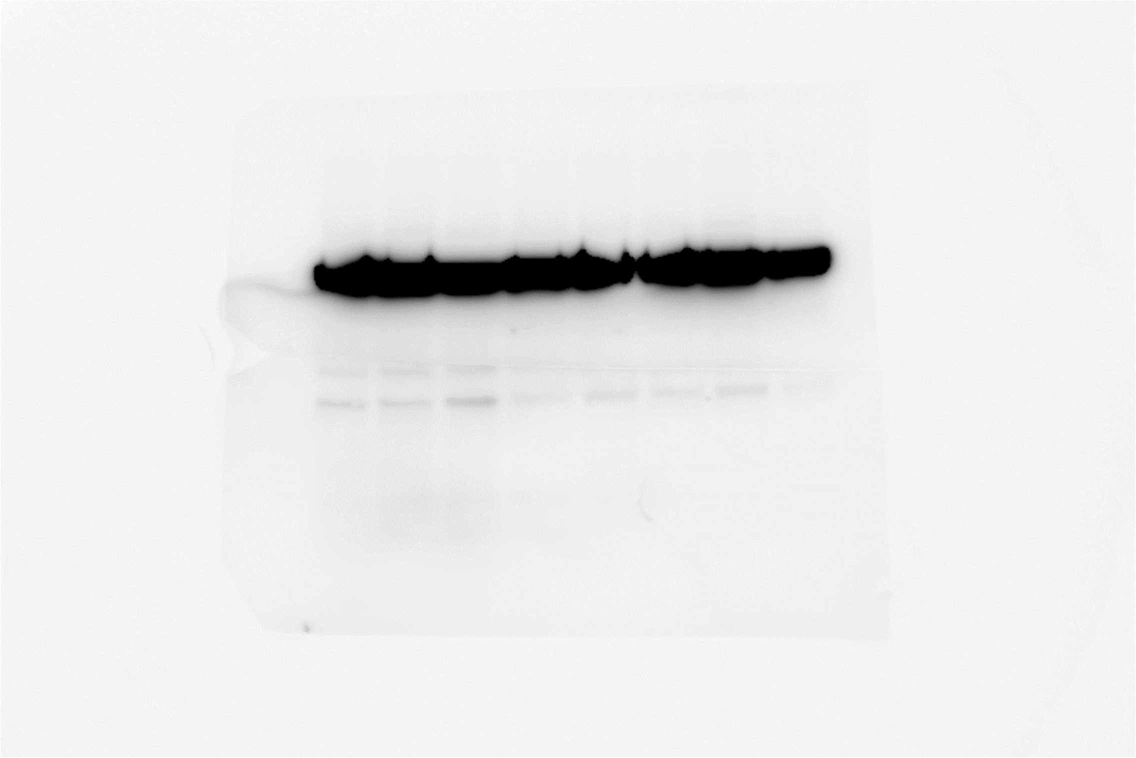

Actin

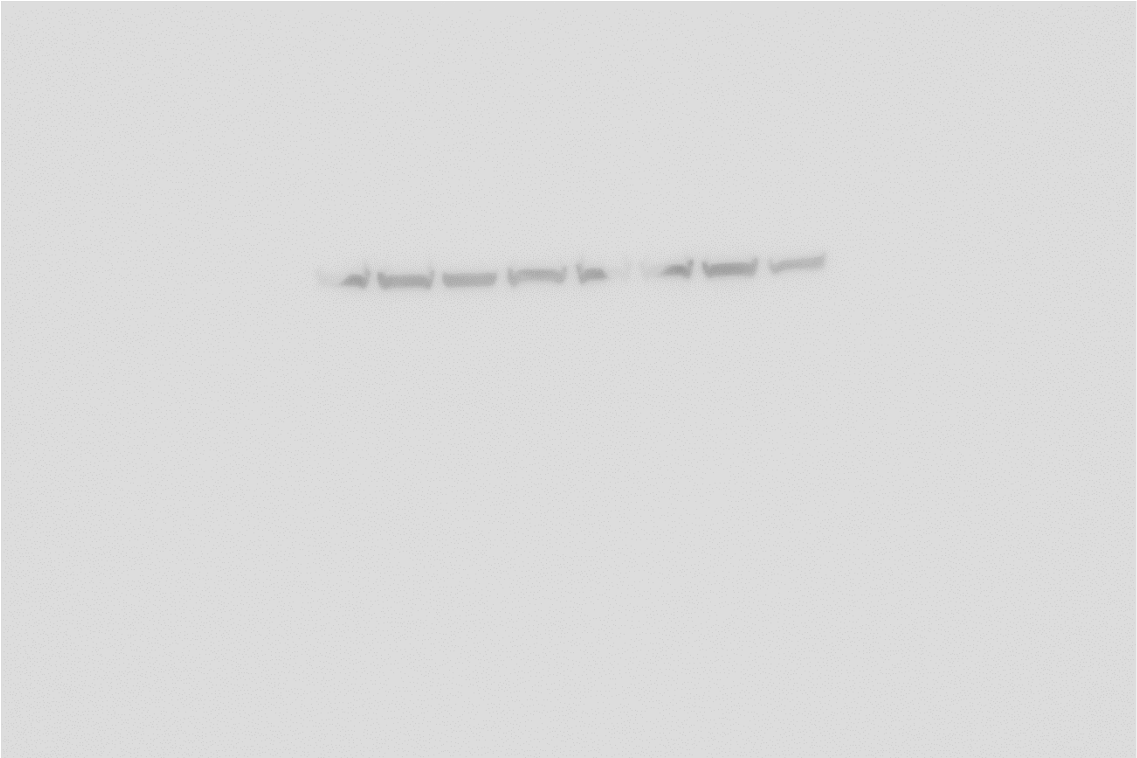

These are the images used for the final figures. All experiments were performed at least three times and every protein was normalized with its own actin.

The authors will provide the uncropped and full-length images upon reasonable request.
